# Quality of life and physician-reported developmental, cognitive, and social problems in children with benign external hydrocephalus—long-term follow-up

**DOI:** 10.1007/s00381-018-4016-2

**Published:** 2018-12-06

**Authors:** Sverre Morten Zahl, Arild Egge, Eirik Helseth, Anne-Britt Skarbø, Knut Wester

**Affiliations:** 1 Department of Clinical Medicine K1, University of Bergen, Bergen, Norway; 2 Department of Ear, Nose and Throat, Aalesund Hospital, N-6026 Aalesund, Norway; 3 Department of Neurosurgery, Oslo University Hospital, Oslo, Norway; 4 Faculty of Medicine, University of Oslo, Oslo, Norway; 5 Department of Clinical Neurosciences for Children, Oslo University Hospital, Oslo, Norway; 6 Department of Neurosurgery, Haukeland University Hospital, Bergen, Norway

**Keywords:** Benign external hydrocephalus (BEH), Quality of life, Neuropsychology, Psychosocial function, Macrocephaly, Outcome studies

## Abstract

**Introduction:**

Benign external hydrocephalus (BEH) is characterized by too rapidly increasing head circumference in infants, combined with typical neuroimaging findings. Psychomotor developmental delay is typically seen during the first few years of life; after that, the children’s development assumedly normalizes. However, little is known about the long-term effects of BEH.

**Methods:**

In this retrospective population-based study, children diagnosed with BEH during the years 1994–2003 in Southern Norway were asked to participate. Included patients (age 8–18 years old) and their parents answered the PedsQL questionnaire. The patient’s family physicians contributed by giving information from medical records, with special emphasis on developmental, cognitive, and social function.

**Results:**

One hundred seventy-six children were identified with BEH. One hundred three patients and 86 parents completed the PedsQL questionnaire. Supplemental medical information for 142 of the patients was received, mainly from their family physicians. Children and adolescents with BEH score themselves better than the normative mean on health-related quality of life, while the parents score their BEH children within the normative mean, except for the school functioning subgroup, where they score significantly lower. Various developmental, physical, and social problems are reported, like mental retardation, speech problems, epilepsy, motor impairment, psychiatric disorders, and cognitive difficulties. Among these patients, there is a discrepancy in some areas between the child-reported and parent-reported quality of life.

**Conclusions:**

Children and adolescents who were diagnosed with BEH during infancy generally do well. However, for some patients, there appear to be various developmental, social, and cognitive problems, and they seem to struggle more in school than their healthy peers.

## Introduction

Benign external hydrocephalus (BEH) is a condition in infants with an incidence of about 0.4 per 1000 live births [30]. It is defined as a rapid increase in the head circumference, typically around the age of 6 months [32]. Radiologically, three neuroimaging features characterize the condition: enlarged subarachnoid spaces—especially overlying the frontal lobes, normal or moderately enlarged ventricles, and a typical widened frontal interhemispheric fissure [2, 13]. Many other symptoms are described, all shared with “ordinary hydrocephalus,” e.g., frontal bossing, dilated scalp veins, hypotonia, and developmental delay. However, these symptoms have been regarded as transient, together with neuroimaging findings. Hence, the condition has been described as being benign, and therefore rarely treated.

Many other terms have been used for the same and similar conditions, such as subdural hygroma/effusion/collection [4, 10, 22], primitive megalencephaly [11], or benign enlargement of the subarachnoid spaces—BESS [15, 27]. For simplicity, the condition will be referred to as BEH in this article.

Few articles have been published on long-term effects of BEH [11, 16, 17]. Only one of these included children who were shunted [16]. Generally, children and adolescents with BEH show subtle neurocognitive difficulties, but the results do vary. Muenchberger et al. reported problems for some patients, especially in school [17]. Laubscher et al. found one patient with mental retardation and several children who were clumsy or delayed in language at school age follow-up [11]. One study found that children with BEH report slightly reduced quality of life [31].

This article is a follow-up and extension of our study from 2017 [16]. We present follow-up information from the patient’s family physicians regarding different problems and diagnoses considered relevant, together with self-reported, and parent-reported health-related quality of life.

## Methods

This is a population-based retrospective study of children diagnosed with BEH during infancy. Medical records of all children referred to two Norwegian university hospitals during the study period (1994–2003) were reviewed and considered for inclusion. These hospitals are the only referral centers for neurosurgery for about 3.34 million people (75% of Norway’s population). For a thorough and relevant description of Norway’s health system, see Wiig et al. [30]. Only children with BEH (increased or increasing head circumference and typical neuroimaging findings) were included. Inclusion criteria were head circumference greater than the 97.5th percentile or crossing two percentiles during the first year of life, and typical neuroimaging findings. From the medical records, information about age, gender, clinical symptoms and signs, neuroimaging, treatment, and follow-up were collected. The patients and their families were invited by a letter to join the study.

Exclusion criteria: a history of head trauma, intracranial hemorrhage, CNS infection, prematurity (birth before 37 weeks of gestation), and other known causes of hydrocephalus.

Consenting patients and parents filled out the Pediatric Quality of Life Inventory (PedsQL) questionnaire. PedsQL is a health-related quality of life measurement tool with good reliability and validity [28], translated and validated for use also in Norway [21]. It generates a total score and further consists of four subscales: physical function, emotional function, social function, and school function. We present raw scores as means and compare them with the normative mean [21]. To be clinically significant, scale score is 70 or lower [9]. Results are presented as raw scores and compared with normative data when available.

In Norway, all inhabitants have been registered with a family physician that provides primary health care and receives reports from medical specialists involved with their patients. We contacted the family physician of every included patient and received medical records. Information from the records about the patient’s health, with an emphasis on developmental, cognitive, and social status, was collected and categorized.

The study was approved by the Regional Committee for Medical Research Ethics.

## Results

One hundred seventy-six children were identified with BEH during the 10-year period. One hundred fifty-two (86.4%) were boys. For further demographic details, see Wiig et al. [30]. Forty-nine (27.8%) of the children received surgical treatment for their hydrocephalus, but information about specific surgical indication was not available for each individual patient. For further information about differences in outcome for treated versus untreated patients, see Mikkelsen et al. [16].

Eighty-eight teenagers (age 13–18 years) and 15 children (age 8–12 years) answered the PedsQL questionnaire. Eighty-six parents completed the corresponding parent version of PedsQL. Table 1 shows the PedsQL scores from the parent (proxy) and self-report questionnaires. They are compared with the normative means [21] using a one-sample *t* test. For the parent reports, only the school score was significantly lower than the normative mean, while the other subscores and total score were lower but not significantly so. The self-reported total scores and all subscores were significantly higher than the normative mean.

**Table 1:** Self- and parent-reported health-related quality of life using the PedsQL questionnaire. Means are compared with normative means using a one-sample *t* test (level of significance *p* < 0.05). For the parent reports, school score is significantly lower than the normative mean. For the self-reports, both total score and all subscores are significantly higher than the normative mean

				One-sample *t* test		
PedsQL parent report	N	Mean (SD)	Min-max	*t* (df)	*p*	Normative mean [21]
Total score	86	83.39 (17.31)	33.70–100	−1.45 (85)	0.150	86.10
Physical health	86	88.83 (16.45)	31.25–100	0.00 (85)	0.998	88.83
Emotions	86	79.83 (20.62)	15.00–100	−0.07 (85)	0.945	79.98
Social	86	83.97 (23.56)	0.00–100	−1.61 (85)	0.112	88.05
School	84	78.27 (20.11)	30.00–100	−4.87 (83)	0.000	88.97
PedsQL self-report	N	Mean (SD)	Min-max	*t* (df)	*p*	Normative mean [21]
Total score	103	89.85 (9.20)	57.61–100	5.04 (102)	0.000	85.29
Physical health	103	93.60 (8.46)	56.25–100	2.98 (102)	0.004	91.12
Emotions	103	85.70 (14.66)	40–100	5.92 (102)	0.000	77.15
Social	103	93.98 (9.27)	60–100	6.42 (102)	0.000	88.12
School	102	83.70 (14.47)	40–100	3.96 (101)	0.000	78.02

When differing between children and teenager PedsQL scores and their respective parents, the results were no different from those reported in Table 1. This applies to both total score and subscores.

For a total of 142 (81%) of the patients, we received medical information from their family physicians. We also received follow-up hospital records for some of the patients. For 38 of these 142 patients, clinically relevant problems were reported. Table 2 summarizes this and shows the corresponding parent- and self-reported PedsQL with mean total scores for those patients where these were available. The number of patients is small, but in general, the parent scores are lower than the self-reported scores, and some scores are also lower than the clinical cutoff score. For the remaining 104 patients (of 142), the physicians/hospitals reported no relevant problems.

**Table 2:** For 142 patients, we received medical information from physicians and hospitals. Thirty-eight of these reported problems/conditions (often more than one) are shown in this table. The table also shows the corresponding PedsQL scores for those patients where we had this information and the amount of shunted patients

Reported problems	No. of patients	Percent of reported patients	No. of shunted patients	Mean PedsQL self-report score (*N*)	Mean PedsQL parent report score (*N*)
Delayed speech	13	9.2%	6/13	84.1 (5)	75.4 (5)
Social behavioral problems	12	8.5%	7/12	85.1 (6)	57.6 (8)
Motor impairment	11	7.7%	5/11	81.0 (6)	75.4 (5)
Mental retardation*	8	5.6%	6/8	94.0 (2)	40.6 (3)
Concentration problems	8	5.6%	2/8	81.5 (7)	75.4 (5)
Cognitive deficits	7	4.9%	4/7	83.4 (4)	48.4 (2)
Epilepsy	7	4.9%	3/7	81.0 (4)	67.6 (5)
ADHD/ADD	6	4.2%	2/6	72.8 (2)	53.3 (3)
Autism spectrum disorders	5	3.5%	1/5	57.6 (1)	57.4 (4)
Anxiety and depression	5	3.5%	0/5	83.7 (3)	68.1 (3)
Dyslexia	4	2.8%	3/4	90.2 (3)	79.9 (2)

*The eight patients with mental retardation are also contained in other groups: speech problems (four of the eight mentally retarded patients); motor impairment (four patients); epilepsy (two patients); autism spectrum disorders (two patients); cognitive deficits (two patients); social behavioral problems (three patients)

Information regarding the 71 patients who did not answer the quality of life questionnaire (self-report nor parent-report) was also explored. Thirty-seven were reported by physicians and hospitals to have no relevant problems. For 17 patients, no supplemental information existed. For the rest (17 patients), various problems were described, as reported in Table 2.

## Discussion

The purpose of this study was to investigate the long-term effects of BEH. Mikkelsen et al. reported that children with BEH show subtle neurocognitive difficulties [16]. Our study population includes some of the same patients. Most children with BEH seem to do quite well during late childhood and adolescence, yet some children report difficulties.

As shown in Table 1, BEH children score within the normative values on the total score on health-related quality of life. Only school-functioning scores were significantly lower than the normative mean, but only by parent reports. In general, the children and adolescents score themselves above the normative mean, and the parents score their children slightly below the normative mean. The results were not different when children (8–12 years) and adolescents (13–18 years) were analyzed separately. It seems that the parent scores better reflect the clinical conditions.

As found in our previous study [16], the only functional area BEH patients seem to struggle on a long-term basis is in school although they do not seem to perceive that themselves. Whether this discrepancy is due to a deficient self-knowledge in children or parental bias towards “expected” problems remains uncertain. There was no difference in PedsQL scores when differing between children and adolescents, and the corresponding parent scores. This observation shows that increasing age not necessarily implies a more “realistic” view on the quality of life.

The divergence in the PedsQL score for school functioning should be the target for future research, for instance with a prospective longitudinal study of this patient group.

Physician-reported problems are shown in Table 2. Some report rather serious conditions, often more than one per child. These patients have no other known causes for their problems. We have no established control group; hence, it is difficult to draw conclusions.

### Mental retardation

The overall prevalence of mental retardation in a Norwegian population study was 6.2/1000 [24]. One previous study describes mental retardation in eight out of 74 patients with BEH and/or megalencephaly [11]. Six of the eight patients with mental retardation in our material had been treated with a shunt. This could reflect a slightly different clinical condition, with more pronounced symptoms leading to shunt surgery, possibly due to a higher ICP, and hence, a larger risk of serious brain damage (mental retardation). Mental retardation is a serious condition that brings about several symptoms. For that reason, some of these patients will also be reported in other groups: speech problems (four of the eight mentally retarded patients); motor impairment (four patients); epilepsy (two patients); autism spectrum disorders (two patients); cognitive deficits (two patients); social behavioral problems (three patients).

### Speech problems

The delayed speech was reported in quite a few of our patients, this has been described in earlier studies, but usually in one or very few patients [3, 8, 19]. Yew et al. reported that six of their 72 patients had verbal deficits detected late during follow-up and not at diagnosis [31]. Unfortunately, we have insufficient information about the degree or duration of speech problems in our study group.

### Motor impairment

Motor impairment/clumsiness was seen in around 6% of patients where we had reliable health information. Additionally, we found that 14 out of the 133 children (10.5%), who later had normal motor development, showed a temporary delay in motor skills typically before 3 years of age. This corresponds well with earlier studies [2, 17, 31]. Delayed gross motor function is described also on a long-term follow-up [8, 18, 19]. For the patients where we also had the quality of life reports, the PedsQL physical subscores did not differ significantly from total score, or between patients and their parents.

### Epilepsy

Epilepsy is reported in some patients. Both seizures during childhood and abnormal electroencephalograms have been reported in infants with BEH [5, 20]. To our knowledge, no earlier studies have reported this as a permanent long-term finding in older children. We do not know the severity of epilepsy in our patients, but the incidence seems higher than in the general population [26].

### Autism spectrum disorders

Autism spectrum disorder was found in five of our patients (all boys). This seems to be a higher incidence than in the general Norwegian pediatric population [25]. The authors of a recent study, using repeated magnetic resonance imaging scans, propose that extra-axial fluid that persists from infancy and beyond 12–24 months of age could be a possible biomarker for the early detection of an autism spectrum disorder risk [23]. Our results may support this possibility.

### Psychiatric disorders

Psychiatric disorders like ADHD (six patients) and anxiety and depression (five patients) have rarely been reported before, probably because very few studies have a long enough follow-up for such symptoms to appear. Muenchberger et al. describe one patient with depression and two with panic attacks, one of them is also diagnosed with hyperactivity [17]. The overall prevalence of mental disorder for this age group in Norway is about 7% [7]. Whether or not our patients were formally diagnosed by specialists are unknown. Based on this and our limited number of patients, we have no reason to suspect that BEH is an important risk factor for developing psychiatric disorders.

### Learning disabilities

Some patients display various learning/cognitive problems (Table 2). To our knowledge, only two studies have reported school functioning in children with BEH. Muenchberger et al. found that eight of 15 patients had to repeat grades or attend special classes [17]. Laubscher et al. found that 11 of 12 children had a normal school outcome [11]. The PedsQL results presented earlier support the belief that BEH is associated with a higher risk of problems in school.

### Social behavioral problems

Social behavioral problems are commonly reported (Table 2). Although unspecific, it seems to have a significant impact on the quality of life, as reported by parents. When looking at the five patients with reported social behavioral problems where we have PedsQL scores from both patients and parents, four of five parents report significantly lower values on the PedsQL social subscores than their children (difference range 15–55). It seems that children with social behavioral problems have reduced self-awareness regarding this. As mentioned earlier, three of these patients were found to be mentally retarded.

When looking at patients with reported PedsQL scores of less than 70, i.e., indicating clinically significant problems for whom supplemental information exists, the medical reports confirm various developmental, cognitive, and social problems.

This study has its limitations. A high number of patients (41%) and parents (51%) did not answer the quality of life questionnaire. This was explored by looking at patients where we did not receive PedsQL answers but did receive supplemental information from family physicians or hospital medical records, and we found quite a few patients with various problems (as reported in Table 2). This shows that our reported PedsQL scores probably are unnaturally high, as conditions like these most likely will cause lower quality of life scores. It is possible that some parents with concern about their child’s development and well-being have preferred not to answer the quality of life questionnaire, and children with difficulties and problems find it too painful. The reason for the difference between child self-reports and parent proxy-reports is debated. It has been demonstrated that levels of agreement can be affected by child age and development, domains investigated, and the parent’s own quality of life [6].

Another limitation is our decision to exclude prematurely born infants and children with subdural hematomas. Prematurity is a risk factor for developing BEH [1, 8]. Subdural hematoma is a known complication to BEH, even without a head trauma [12, 14, 29]. In retrospect, we believe this deprived us of patients who could have enlightened our knowledge about long-term effects.

## Conclusions

Patients with BEH generally seem to do well as they grow up. They report a normal quality of life except for school functioning were some of the children seem to struggle more than their peers do. In addition, various medical, social, and cognitive problems are reported for some of the patients.
